# PET-derived heteroatom-doped carbon quantum dots as color-modulated solid-state fluorescent materials†

**DOI:** 10.1039/d5ra02014j

**Published:** 2025-05-06

**Authors:** Peerapong Promcharoen, Peerapong Chumkaeo, Sunichaya Charoenchaidet, Sumate Charoenchaidet, Ekasith Somsook

**Affiliations:** a NANOCAST Laboratory, Center for Catalysis Science and Technology (CAST), Department of Chemistry, Center of Excellence for Innovation in Chemistry, Faculty of Science, Mahidol University 272 Rama VI Rd., Ratchathewi Bangkok 10400 Thailand ekasith.som@mahidol.ac.th; b Triam Udom Suksa School 227 Phaya Thai Rd., Pathum Wan Bangkok 10330 Thailand; c SCG Chemicals Co. Ltd. 1 Thanon Siam Cement, Bang Sue Bangkok 10800 Thailand

## Abstract

Plastic waste was transformed into high-performance quantum dots (QDs), combining technological innovation with a focus on environmental sustainability. The excellent fluorescence properties of the synthesized quantum dots were utilized to detect Fe^3+^ and F^−^ ions with high sensitivity and selectivity in an “on–off–on” dual-mode fashion. Additionally, the synthesized quantum dots exhibited stable solid-state fluorescence, enabling their use in solid-phase applications without the typical fluorescence loss observed in other materials. The versatility and tunability of the synthesized materials were demonstrated by producing three different emission colors, achieved through the incorporation of various heteroatoms during the synthesis process. This solid-state fluorescent material provides a pathway for sensing and optoelectronic applications, as well as advanced optical devices with customizable designs in the future.

## Abstract 2

High-performance quantum dots (QDs) derived from PET waste for the detection of Fe^3+^ and F^−^ ions with high sensitivity and selectivity in an “on–off–on” dual-mode fashion.

## Graphical abstract

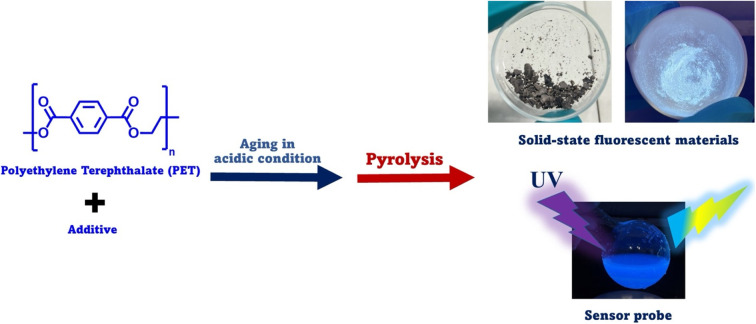

## Introduction

Plastic waste has become a significant concern for both the environment and human health. Globally, billions of tons of plastic waste are discarded annually. As non-biodegradable materials, plastics contribute to land and water pollution and pose a serious threat to living organisms.^1–3^ Addressing the plastic waste crisis requires innovative recycling and waste management strategies that not only mitigate environmental impacts but also transform waste into valuable chemicals.^4–8^ Recent innovations have explored methods for transforming plastic waste into valuable functional materials.^9^ One promising solution is the recycling of plastic waste into quantum dots (QDs) and related materials, which have applications in nanocrystalline semiconductors due to their outstanding photovoltaic and electrical properties.^10–13^ This approach not only addresses plastic waste but also yields materials with significant technological potential. Furthermore, their synthesis generates only small, non-toxic by-products, which are removed during purification.^14–16^

Quantum dots (QDs) exhibit several key features, including broad absorption, excellent photostability, and size-dependent emission at specific wavelengths.^17,18^ Based on these properties, QDs are suitable for applications in bioimaging, photovoltaics, drug delivery, and, primarily, fluorescence sensing.^19–24^ Moreover, the exceptional selectivity of QDs for various target molecules is attributed to their quantum confinement effects and colloidal chemistry, which are influenced by the aforementioned properties.

Among the diverse types of QDs, carbon-based quantum dots (CQDs) have emerged as a sustainable and environmentally benign alternative to traditional metal-based QDs.^25^ Compared to widely studied metal-based QDs such as CdSe, PbS, and InP, CQDs offer significant advantages, including lower toxicity, enhanced aqueous solubility, ease of functionalization, and excellent biocompatibility.^26–29^ Furthermore, CQDs eliminate the use of heavy metals that pose ecological and health risks, thereby offering a safer platform for applications in environmental and biological sensing.

In sensing applications, the fluorescence properties of QDs are utilized, as their fluorescence emission can be modulated through interactions with target molecules.^30,31^ This modulation is harnessed in highly sensitive and selective fluorescence sensors capable of detecting extremely low concentrations of analytes.^32–35^ The potential of utilizing QDs in sensing applications can enhance environmental monitoring, disease diagnosis, and the regulation of various processes.^36–38^

Monitoring Fe^3+^ and F^−^ ions is essential due to their significant environmental and health impacts.^39–43^ The concentration of Fe^3+^ ions in water can be harmful, indicating potential water pollution and posing risks to water quality and the health of aquatic life.^44^ Similarly, while F^−^ ions are beneficial in small amounts, excessive concentrations can be harmful, making accurate monitoring of their levels essential.^45^ Traditional detection methods are often constrained by limitations in sensitivity, selectivity, and operational complexity.^46,47^

Producing QDs from plastic waste represents a remarkable integration of waste recycling and the creation of innovative materials.^48–52^ This study focuses on synthesizing QDs from plastic waste and utilizing them as on–off–on fluorescence probes for detecting Fe^3+^ and F^−^ ions. Consequently, these QDs serve as efficient multifunctional sensors for both metal ions and anions, playing a vital role in environmental monitoring.

## Experimental

### Chemicals and reagents

Cysteine, nickel(ii) chloride anhydrous, and melamine monomer were purchased from Tokyo Chemical Industry Co., Ltd. (Tokyo, Japan). Cobalt(iii) chloride, magnesium nitrate, scandium(iii) chloride, and sodium bromide were purchased from Sigma-Aldrich (Germany). Zinc acetate dihydrate and sodium nitrate were purchased from Merck (Germany). Iron(ii) chloride tetrahydrate and sodium acetate were purchased from Fluka (Switzerland). Di-sodium hydrogen orthophosphate dodecahydrate (Na_2_HPO_4_·12H_2_O) was purchased from Ajax Chemical Ltd. (Australia). Potassium iodide was purchased from Carlo Erba. Hydrochloric acid (36.5%), orthophosphoric acid, sodium chloride, and sodium bicarbonate were obtained from Labscan (Thailand). Stock cation and anion solutions were prepared at 1000 mg L^−1^ in ultrapure water and stored at 25 °C. Ultrapure water (18.2 MΩ cm at 25 °C) was utilized throughout all experiments.

### Instrumental

The optical properties were determined using UV-vis spectroscopy and a fluorescence spectrophotometer. UV-vis absorption spectra were obtained using an Avaspec 2048 spectrometer (Apeldoorn, the Netherlands). Fluorescence spectra were obtained using a Cary Eclipse fluorescence spectrophotometer manufactured by Agilent (California, United States). To study morphological characteristics, high resolution transmission electron microscopy (HRTEM) was conducted using a JEOL JEM-2010 microscope (Japan). The accelerating voltage was 120 kV. To prepare the samples, the synthesized quantum dot samples were ultrasonically dispersed and applied to a Formvar-coated copper grid. A scanning electron microscope (SEM) analysis was conducted using a JSM-5200 scanning electron microscope (JEOL, Tokyo, Japan). FT-IR spectra were obtained using the diffuse reflectance technique with a BX FT-IR spectroscope (PerkinElmer, MA, USA). The powder of the synthesized quantum dots was analysed by X-ray powder diffraction (XRD). The patterns were recorded at a scanning angle (2*θ*) of 5–70° using a 2nd Generation D2 Phaser Powder X-ray Diffractometer, which was supplied by Bruker Corporation (Germany). An X-ray photoelectron spectrometer was used to analyze the chemical composition of the sample's surface. The elemental compositions within the materials were investigated by X-ray Photoelectron Spectroscopy (XPS) using ACXIS ULTRADLD, Kratos Analytical, Manchester, UK. The analysis chamber was sustained at an initial pressure of around 5 × 10^−9^ torr. The specimens were energized using a 700 × 300 μm spot area X-ray hybrid mode, employing monochromatic Al Kα_1,2_ radiation at 1.4 keV. The X-ray anode functioned at a voltage of 15 kV, a current of 10 mA, and a power output of 150 watts. A hemispherical analyser was used to detect photoelectrons oriented at an angle of 45° to the normal of the sample surface.

### Preparation of nitrogen-doped carbon quantum dots (NCQDs)

NCQDs were prepared by aging 1.50 g of repurposed PET bottle (0.5 mm × 0.5 mm) and 1.2 g of melamine monomer in 15 cm^3^ of HCl acid (36.5%) at 150 °C for 1 hour. Then, the mixture was transferred to the crucible, and the mixture was heated to 280 °C for 5 minutes in the furnace under atmospheric air. After the sample was cooled to room temperature, the mixture was neutralized with 1.0 mol per dm^3^ NaOH until the pH reached 7.0. The mixture was then filtrated and washed with ultrapure water. The solid product was dried in an oven at 100 °C overnight to obtain NCQDs as the final product. Before using the solution fluorescence sensor, the solid product was redissolved using 1.0 mol per dm^3^ NaOH, and the salt ions in the solution were further removed using a dialysis bag for 12 hours.

### Preparation of nitrogen and sulfur-doped carbon quantum dots

Cysteine (1.2 g) was used as the source of heteroatoms (nitrogen and sulfur) to age with the repurposed PET bottle. Then, NSCQDs were prepared by applying the same method mentioned above.

### Preparation of boron-doped carbon quantum dots

Sodium borate (1.2 g) was used as the source of heteroatoms (boron) to age with a repurposed PET bottle. Then, BCQDs were prepared by applying the same method mentioned above.

### Preparation of phosphorus-doped carbon quantum dots (PCQDs)

PCQDs were prepared by aging 1.50 g of a repurposed PET bottle (0.5 mm × 0.5 mm) in 15 cm^3^ of orthophosphoric acid at 150 °C for 1 hour. Then, the mixture was transferred to the crucible, and the mixture was heated to 280 °C for 5 minutes in the furnace under atmospheric air. After the sample was cooled to room temperature, the mixture was neutralized by 1.0 mol per dm^3^ NaOH until the pH reached 7.0. The mixture was then filtrated and washed with ultrapure water. The solid product was dried in an oven at 100 °C overnight to obtain PCQDs as the final product. Before using the solution fluorescence sensor, the solid product was redissolved using 1.0 mol per dm^3^ NaOH, and the salt ions in the solution were further removed using a dialysis bag for 12 hours.

### Fluorescence measurements

Fluorescence measurements were conducted using the subsequent methodology. A solution of as-synthesized nanofluorescence probes was prepared by dissolving NCQDs (NSCQDs, BCQDs or PCQDs) nanoprobes in a potassium-phosphate buffer at pH 7.0. Subsequently, 300.0 μL of the solution was combined with 100.0 μL of supplementary potassium-phosphate buffer and 100.0 μL of Fe^3+^ ion standard solution without incubating the samples. The fluorescence intensity of the mixed solution was measured and collected from 330 to 550 nm while maintaining a constant excitation wavelength. The slit width was set at 10 nm, and the experiment was performed at room temperature (25 °C).

To complete the fluorescence measurement of the “off–on” stage, 300.0 μL of the NSCD nanoprobe solution was combined with 100.0 μL of Fe^3+^ ion standard solution. Then, 100.0 μL of the F^−^ ion standard solution was added. The fluorescence intensity of the mixed solution was measured and collected without an incubation time.

## Results and discussion

**Fig. 1:**
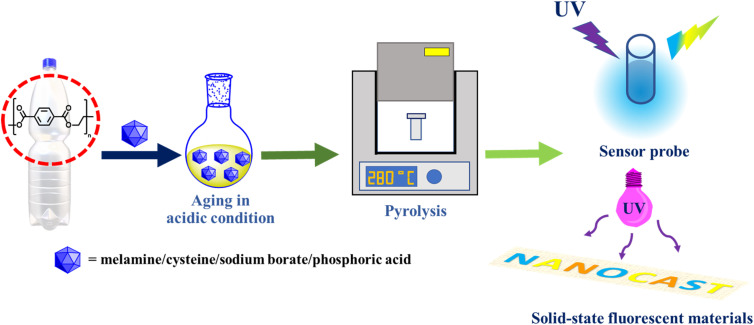
Schematic of the synthesis and multifunctional behaviours of heteroatom-doped QDs derived from repurposed PET bottles.

Heteroatom doping is an efficient technique for synthesizing quantum dot particles with enhanced optical and chemical characteristics by modifying their carbon frameworks and chemical configurations. Four varieties of heteroatom-doped quantum dots, namely N-doped, N,S-doped, B-doped, and P-doped, were effectively synthesized using acid-catalyzed cleavage of a recycled PET bottle precursor utilizing hydrochloric acid (HCl) or phosphoric acid as catalysts for oligomer synthesis. The product, along with sources of heteroatoms, was placed in a crucible and calcinated at 280 °C to facilitate the full transformation of all precursors into the desired products. Fig. 1 depicts the synthesis process for four varieties of heteroatom-doped quantum dots (QDs). The products generated from each calcination procedure were characterized using several necessary equipment to verify their qualities.

**Fig. 2:**
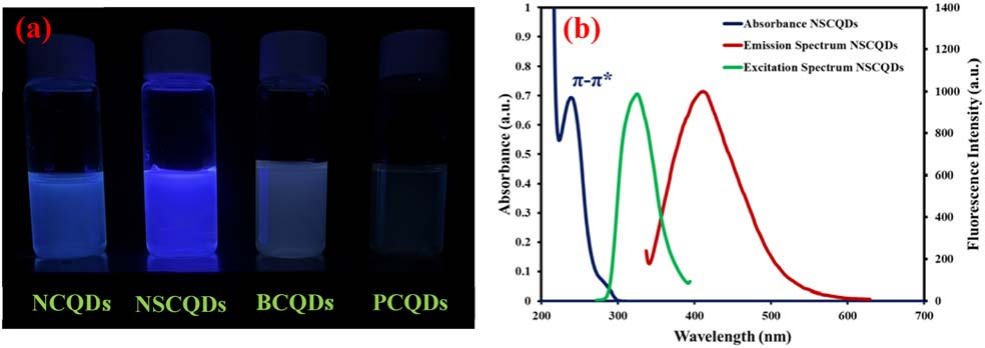
(a) Photograph of synthesized NCQDs, NSCQDs, BCQDs, and PCQDs. (b) Optical spectra profile of the synthesized NSCDs (absorbance (dark blue), excitation spectrum (green), and emission spectrum (dark red)).

The optical properties of the synthesized quantum dots were extensively characterized using UV-vis absorption and fluorescence spectroscopy to assess their potential for use in fluorescence-based applications. The UV-vis spectra also displayed sharp absorption peaks, suggesting the existence of quantum confinement effects common to well-resolved and defined quantum dots. However, these peaks were found between 200 and 400 nm depending on the conditions under which the quantum dots were synthesized and on the source of the heteroatoms incorporated into the quantum dots. The position and sharpness of the absorption edges additionally evidenced homogeneous particle size distribution; thus, the controlled synthesis method was used to transform plastic waste into a functional material. The optical behaviour of the synthesized QDs was further explored with fluorescence spectroscopy. When quantum dots were excited at specific wavelengths, corresponding to their absorption maxima noted in the UV-vis spectra, a strong and stable emission was recorded (Fig. 2a).

The optimal excitation wavelength of the NSCQDs is 327 nm according to the excitation spectrum illustrated in Fig. 2a. In addition, the absolute fluorescence quantum yields of NCQDs, NSCQDs, BCQDs, and PCQDs were measured by the integrating sphere attachment, and the results were as high as 18.4%, 34.4%, 23.5%, and 10.2%, respectively. Among these, NSCQDs have attracted remarkable attention owing to their potential analytical and biological applications. They display excellent optical and sensing properties in aqueous media. Furthermore, using boron as a source of heteroatom, it turned out that the emission wavelength shifted after the pH of the solution was changed (Fig. S1†), which suggests that BCQDs have some limitations for use as a potential sensor probe in the future.

**Fig. 3:**
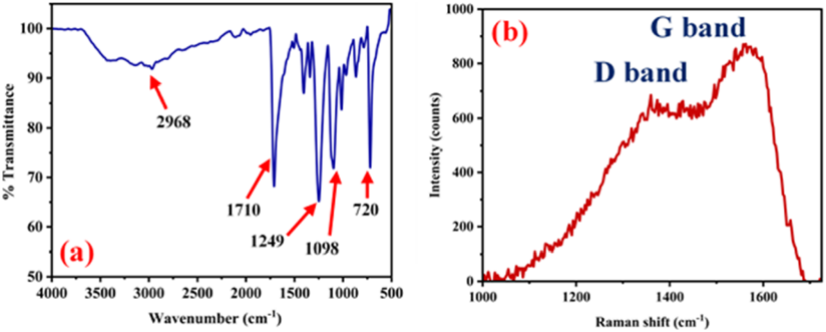
(a) FTIR spectrum. (b) Raman spectrum of the synthesized NSCQDs.

**Fig. 4:**
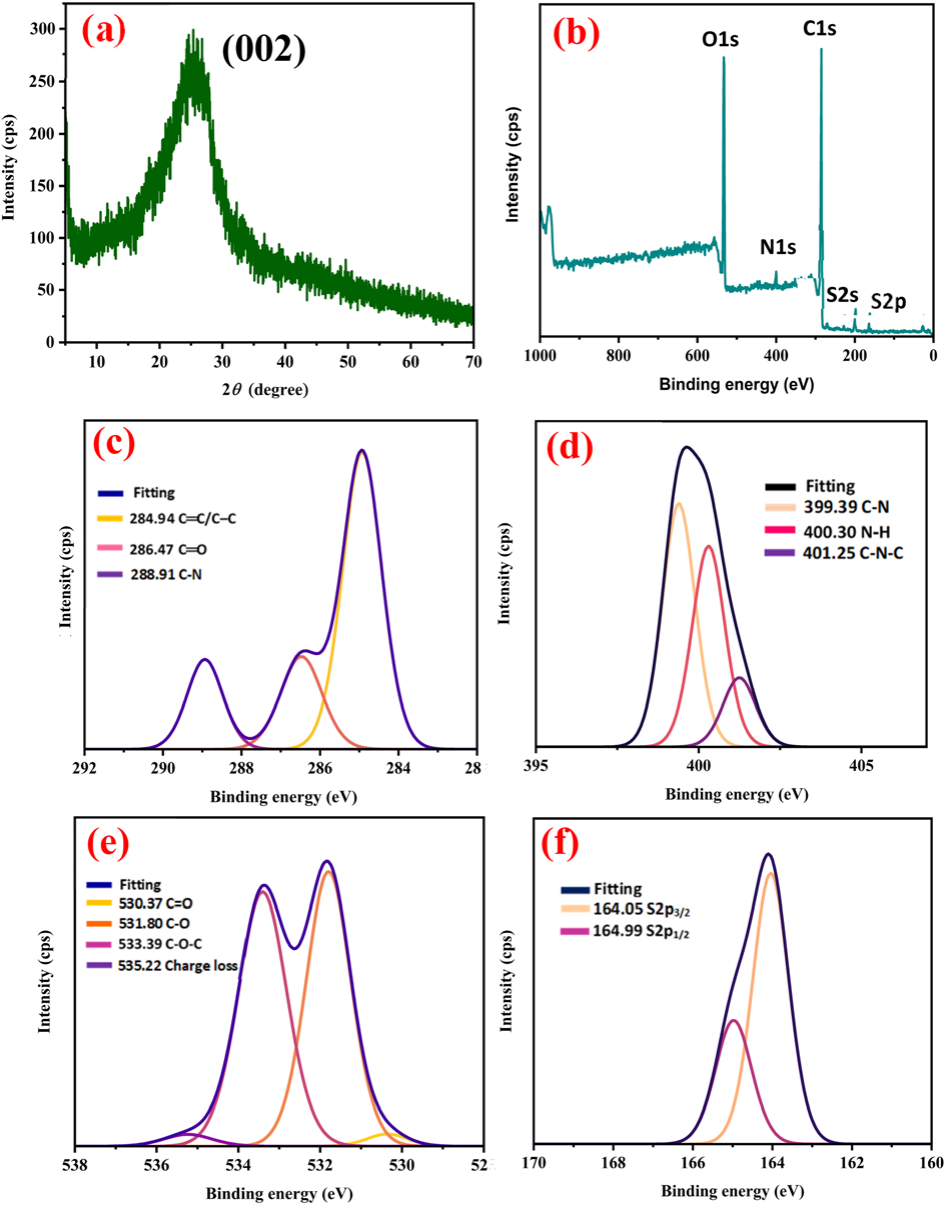
(a) XRD diffractogram of the synthesized NSCQDs. (b) XPS survey spectrum of NSCQDs. Detailed (high-resolution) scans for (c) C 1s, (d) N 1s, (e) O 1s, and (f) S 2p spectra of NSCQDs.

Next, structural investigations were performed. The possible chemical bonds and surface functional groups of NSCQDs were thoroughly investigated by infrared spectroscopy (FTIR), X-ray photoelectron spectroscopy (XPS) and Raman spectroscopy. The FTIR spectrum of NSCQDs is shown in Fig. 3a. NSCQDs exhibited low broad absorption bands centered at 3420 cm^−1^ attributed to the stretching vibrations of the N–H and O–H groups. The observed band at 1710 cm^−1^ corresponds to the occurrence of the C=O group of carboxylic acid or the presence of the stretching C=O group of amide. Additionally, the presence of C–O on NSCQDs was confirmed by the band appearing at 1249 cm^−1^, while the characteristic peaks at 1098 cm^−1^ indicated the existence of the C–S group.^53^ It was demonstrated according to the FTIR that NSCQDs contain abundant functional groups, such as pyrrole, N–H, CO–NH and C–S, depending on the raw materials. The disordered (D) band at 1360 cm^−1^ is related to the presence of sp^3^ defects, while the crystalline (G) band at 1560 cm^−1^ is attributed to the in-plane vibration of sp^2^ carbons.^54,55^ The ratio of intensities (*I*_D_/*I*_G_) is found to be 0.79 for NSCQDs, which means that NSCQDs are highly graphitic materials. In addition, the broad peak at 2*θ* = 25.3° shown in the XRD pattern (Fig. 4a) is the (002) plane of graphene, which is in good agreement with the results obtained from the Raman spectra.

XPS is also used to analyze the structure of NSCQDs. The full spectrum of XPS is shown in Fig. 4b, and it can be found that there are four typical binding peaks at 164, 28, 400, and 533.0 eV, representing the binding energy peaks of S 2p, C 1s, N 1s, and O 1s, respectively, that is, the synthesized NSCQDs mainly contain C, N, S, and O elements. The survey analysis indicated the successful doping of nitrogen for the NSCQDs. It can be found that the high-resolution C 1s spectrum can be fitted with three peaks at 284.9, 286.5, and 288.9 eV, representing C=C/C–C, C=O, and C–N bonds, respectively.^53^ In addition, the presence of C=O bonds can be observed in the O 1s spectrum (Fig. 4e and S2†).

Moreover, the deconvoluted spectrum of S 2p shows two peaks of sulfur at 164.05 and 164.99 eV (Fig. 4f), belonging to the spin–orbit coupling of thiophene S 2p_3/2_ and S 2p_1/2_, respectively.^53,56^ The above results agree well with the results of the FTIR analysis. In summary, the as-prepared NSCQDs were not only successfully doped with nitrogen in the form of N-pyrrole structures but were also covered with –NH_2_ and abundant oxygen-containing functional groups, making it easy to combine with various materials for modification and functionalization in the form of chemical bonds. To complete the investigations of the structures, other heteroatom-doped quantum dots were characterized successfully, as shown in Fig. S3–S8.† The NCQDs exhibited diffraction characteristics similar to graphene structures, while the BCQDs and PCQDs showed distinct sharp peaks, indicating mixed-phase crystalline domains.

**Fig. 5:**
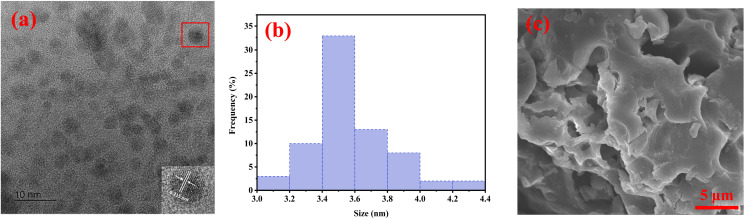
(a) HRTEM image of NSCQDs, (b) size distribution graph of NSCQDs corresponding to (a), and (c) typical SEM image of NSCQDs.

For morphological characteristics of NSCQDs, the as-prepared NSCQDs were characterized by high resolution transmission electron microscopy (HRTEM). The results are shown in Fig. 5a. It can be observed that NSCQDs are spherical and well dispersed, and there is no agglomeration.

The diameter of the as-prepared NSCQDs ranges from 3.0 to 4.4 nm with an average size of 3.6 nm (Fig. 5b). Furthermore, for the morphological study, FESEM images were taken, showing a particle agglomeration-like porous cavity (Fig. 5c). It was also observed that NSCQDs might be converted into larger clusters.

### Quantitative determination of NSCQDs

**Fig. 6:**
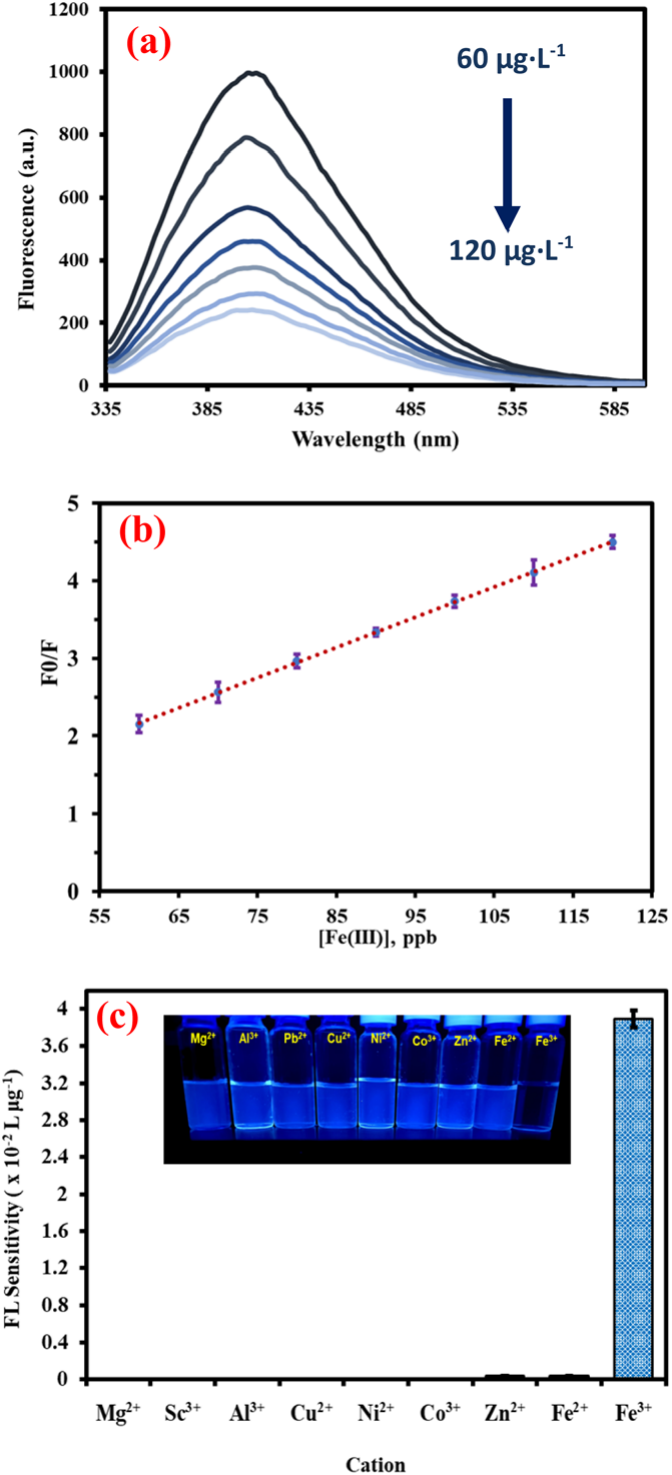
(a) Fluorescence spectra of NSCQDs in the presence of different concentrations of Fe^3+^ ions, (b) calibration curve of NSCQDs in the presence of different concentrations of Fe^3+^ ions, (c) selectivity of NSCQDs sensing probe in the presence of different types of cations.

As fluorescence sensor probes, the synthesized NSCDs showed remarkable efficiency in the detection of Fe^3+^ ions. Under optimal conditions, a robust detection capability down to low analyte concentrations was indicated by a fluorescence response that had a precise linear relationship with the concentration of Fe^3+^ ions in the range of 60–120 μg L^−1^ (Fig. 6a). The calibration equation was obtained as follows: [(*I*_0_/*I*) − 1] = (0.0389 ± 0.0002)*C*_[Fe(III)]_ − (0.1672 ± 0.028), where *R*^2^ = 0.9998 (Fig. 6b). At 34.4%, the measured quantum yield emphasizes the good photoluminescent properties of the material, which are pertinent to fluorescence-based sensing. In the presence of Fe^3+^ ions, a distinct and repeatable, consistent quenching effect was observed with a drop in fluorescence intensity. The quenching behavior can be ascribed to the effective interaction between the Fe^3+^ ions and the surface of the quantum dots, especially those introduced by the N and S dopants. Specifically, Fe^3+^ ions possess a high affinity toward electron-donating groups, such as surface amino (–NH_2_), thiol (–SH), or carboxyl (–COOH) moieties, commonly present in N,S-doped carbon-based QDs. The coordination of Fe^3+^ with these surface sites can lead to non-radiative electron or energy transfer, effectively quenching the excited-state fluorescence.^57^

A comprehensive evaluation of the selectivity of the probe in the presence of different competing cations is based on a fluorescence sensor probe developed using an established efficiency to detect Fe^3+^ ions. We further investigated the selectivity of the as-synthesized quantum dots and observed a pronounced and distinct response of fluorescence quenching to Fe^3+^ ions, but not to other cations, including Mg^2+^, Sc^3+^, Al^3+^, Cu^2+^, Ni^2+^, Co^3+^, Zn^2+^ or Fe^2+^ (Fig. 6c). These observations emphasize the selective quenching properties of the sensor probe, indicating unusually high specificity, as the Fe^3+^ ions could discriminate in an interfering mixture of ions.

To better verify the probe's selectivity, fluorescence sensitivity measurements were carried out in the presence of various cations, and Fe^3+^ ions showed the highest fluorescence sensitivity. This high selectivity can be attributed to the unique surface chemistry of the synthesized quantum dots, which promote a stronger and more favourable interaction with Fe^3+^ ions compared to other cations.

**Fig. 7:**
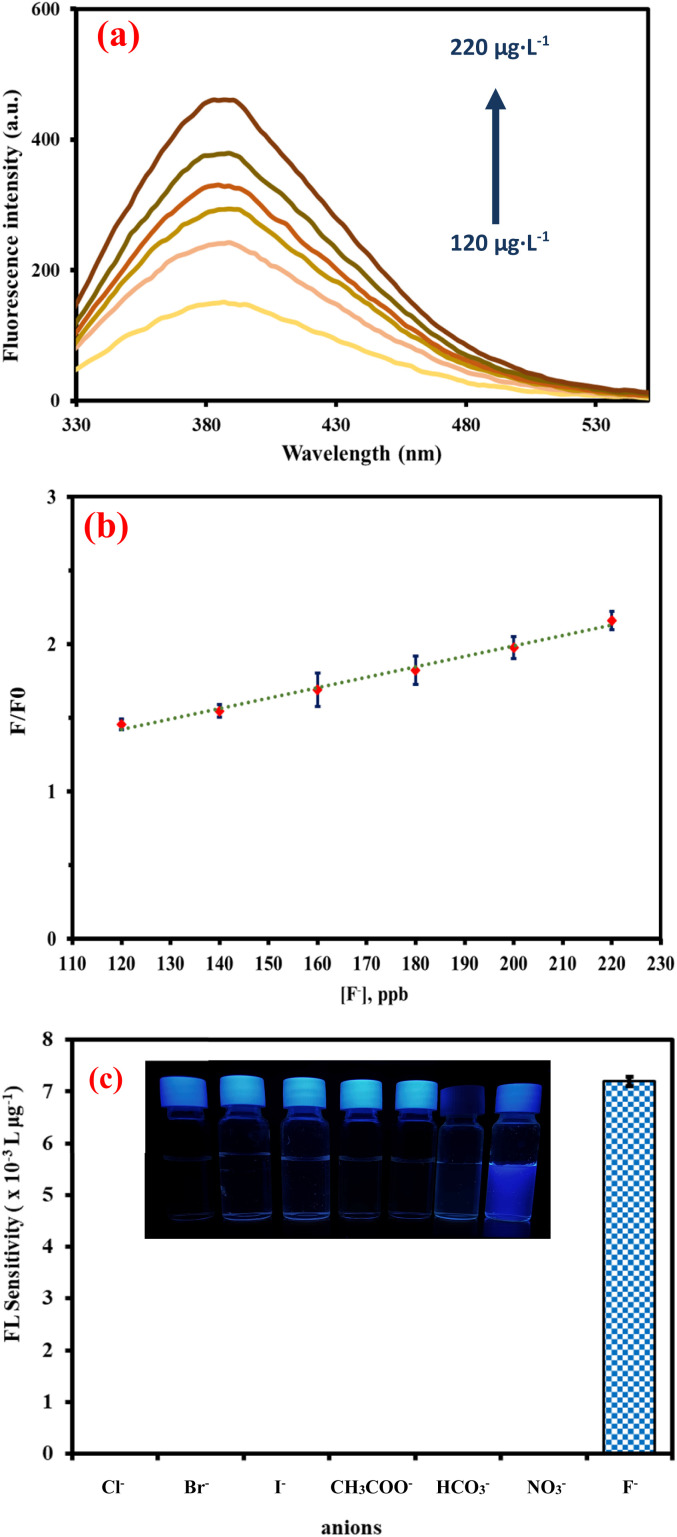
(a) Fluorescence spectra of NSCQDs–Fe^3+^ in the presence of different concentrations of F^−^ ions, (b) calibration curve of NSCQDs–Fe^3+^ in the presence of different concentrations of F^−^ ion, (c) selectivity of NSCQDs–Fe^3+^ sensing probe in the presence of different types of anions.

Next, the feasibility of using the complex between the synthesized quantum dots and Fe^3+^ ion (NSCQDs–Fe^3+^) as a fluorescence sensor probe for the detection of fluoride anion was also evaluated using an “off–on” fluorescence mechanism. Although fluorescence quenching was observed in higher Fe^3+^ ion concentrations, the addition of fluoride anions led to a drastically increased fluorescence intensity, turning the system from being “off” to “on”. The reopening of the fluorescence signal observed in this revival of fluorescence indicates a particular interaction with the Fe^3+^ iron *via* the fluoride anions, which release free NSCQD quantum dots. The probe had a linear response distinct to fluoride concentrations in the range of 120–220 μg L^−1^, with a sensitivity of 0.0072 L μg^−1^, allowing for the highly accurate detection of low concentrations of fluoride (Fig. 7a and b). To confirm the formation of a new product resulting from the interaction between Fe^3+^ and F^−^ ions during the fluorescence recovery (“on”) stage, X-ray diffraction (XRD) analysis was performed on the resulting mixture. The XRD pattern exhibited distinct new peaks, indicating the formation of a crystalline Fe–F species, such as iron(iii) fluoride or related coordination complexes. This structural evidence supports the hypothesis that the recovery of fluorescence is associated with the removal of Fe^3+^ from the quantum dot surface *via* precipitation or complexation with F^−^ ions, effectively restoring the emissive state of the quantum dots (Fig. S9†).

The next studies were carried out to assess the selectivity of the probe towards fluoride anions, including, Cl^−^, Br^−^, I^−^, CH_3_COO^−^, HCO_3_^−^, and NO_3_^−^ ions (Fig. 7c). The results showed that the fluorescence intensity responded significantly to fluoride anions, while other anions resulted in no change in fluorescence intensity, or very low. The selective ‘off–on’ response of the Fe^3+^ ions to the selective reaction with the anions is attributed to the interactions of F^−^ with the Fe^3+^ ions that preferentially bind F^−^ over other anions.

These results demonstrate the dual functional aspect of these synthesized QDs, which can be utilized as versatile fluorescence sensors that can detect both cations and anions with unique fluorescence responses. The adaptability of the synthesized material to detect fluoride in complex samples proves to be a promising candidate for detecting fluoride where high selectivity is demanded. Furthermore, the success of this “off–on” sensor mechanism demonstrates the potential of plastic waste to be transformed into an environmentally benign and efficient material for use in advanced sensing technologies.

### Application of heteroatom-doped quantum dots as solid-state fluorescence materials

**Fig. 8:**
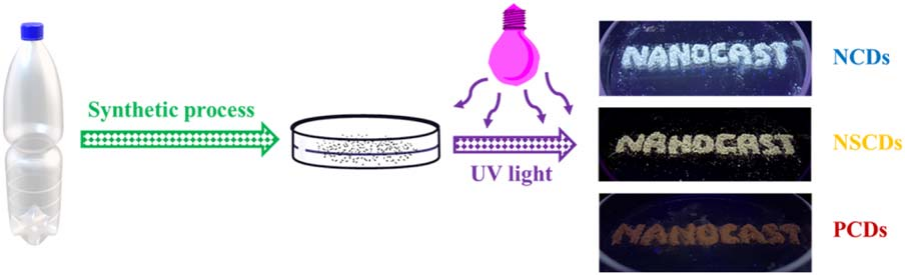
Solid-state fluorescence of synthesized quantum dots under UV light, showcasing three distinct emission colours:NCQDs, NSCQDs, and PCQDs.

One intriguing feature of the synthesized quantum dots is that they can display solid state fluorescence similar to the solid material in all solid form and exposed to UV light (Fig. 8). This was especially visible when quantum dots were dried and illuminated with UV, which energetically showed bright and stable luminescence. Synthesized NCQDs, NSCQDs, and PCQDs also show strong emission fluorescence spectra with a maximum in the solid state. The corresponding fluorescence spectra are depicted in Fig. S10–S12.† In contrast, the synthesized quantum dots contained fluorescence intensity and color consistency, unlike some traditional fluorescent materials, many of which are sensitive to aggregation-induced quenching in the solid state. The stability of the fluorescence, however, suggests that the surface chemistry of the quantum dots effectively prevents the typical solid-state fluorescence loss observed with aggregation.

The next advantages of this work are that it is the successful solid-state synthesis of QDs and that it is possible to fabricate three distinct solid state QD colors. The tunable optical properties of this material were demonstrated by growing it using different sources of heteroatoms in synthesis, which allowed us to tune the emission color across the spectrum. The ability to use controlled heteroatom incorporation to engineer the color of synthesized material broadens the functional scope of the material and opens the door to the development of customizable optical devices. *Via* solid-state fluorescence, the synthesized material exhibits excellent robustness, which not only provides a greater utility in solution but also the possibility for use as a solid-phase sensor, optoelectronic device, or display material.

## Conclusions

In this work, we successfully transformed PET plastic waste into high performance quantum dots that offered a potential sustainable route to develop advanced fluorescent materials. We showed that the synthesized quantum dots had excellent sensitivity and selectivity for detecting Fe^3+^ and F^−^ ions through distinct “on–off–on” fluorescence responses. Additionally, the quantum dots maintain their fluorescence in solid form, indicating that the synthesis method for creating them from plastic waste is highly versatile and robust. Here, the synthesis of the solid-state fluorescence quantum dots enabled the fabrication of three different emission colors utilizing different sources of heteroatoms to extend the material's use for diverse sensing applications, optoelectronics and display technologies. Overall, this study not only points to the potential for the material aspects of waste materials to be transformed into useful resources for environmentally friendly synthesis methods but also to further uses in fluorescence-based technologies.

## Data availability

The data supporting the findings of this study are available from the corresponding author upon request.

## Author contributions

Peerapong Promcharoen: writing – original draft, visualization, validation, methodology, formal analysis. Peerapong Chumkaeo: writing – original draft, visualization, validation, methodology, formal analysis. Sunichaya Charoenchaidet: validation, methodology, formal analysis. Sumate Charoenchaidet: supervision, project administration, conceptualization. Ekasith Somsook: supervision, project administration, methodology, investigation, conceptualization.

## Conflicts of interest

There are no conflicts to declare.

## Supplementary Material

RA-015-D5RA02014J-s001
