# Supplementary material for: PET-derived heteroatom-doped carbon quantum dots as color-modulated solid-state fluorescent materials
Source: RSC Adv. 2025 May 6;15(18):14420–7. doi: 10.1039/d5ra02014j (PMC12053552; doi:10.1039/d5ra02014j)
Supplement: RA-015-D5RA02014J-s001 [file RA-015-D5RA02014J-s001.pdf]

Supplementary Information (SI) for RSC Advances.
This journal is © The Royal Society of Chemistry 2025

PET-Derived Heteroatom-Doped Carbon Quantum Dots as Color-Modulated Solid-State Fluorescent Materials

*Peerapong Promcharoen,[1] Peerapong Chumkaeo,[1] Sunichaya Charoenchaidet,[2] Sumate Charoenchaidet,[3] Ekasith Somsook[1]**

[1]NANOCAST Laboratory, Center for Catalysis Science and Technology (CAST), Department of Chemistry and Center of Excellence for Innovation in Chemistry, Faculty of Science, Mahidol University, 272 Rama VI Rd., Ratchathewi, Bangkok 10400, Thailand

[2]Triam Udom Suksa School, 227 Phaya Thai Rd., Pathum Wan, Bangkok 10330, Thailand

[3]SCG Chemicals Co.Ltd., 1 Thanon Siam Cement, Bang Sue, Bangkok 10800, Thailand

E-mail: ekasith.som@mahidol.ac.th

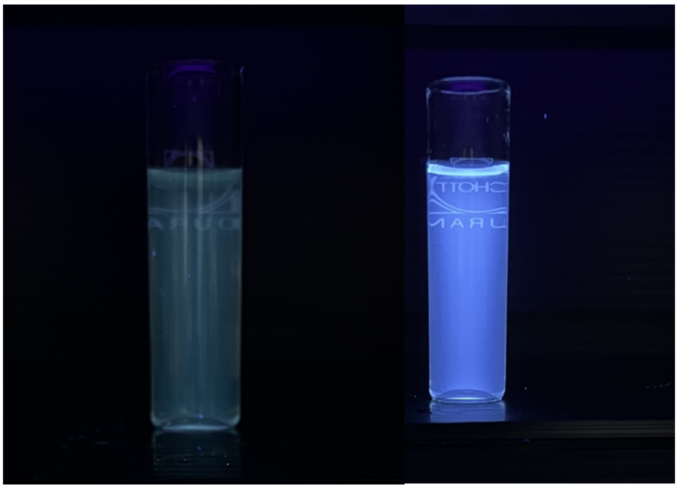

Figure S1. Photograph of synthesized **BCQDs** in the acidic condition (left) and basic condition (right).

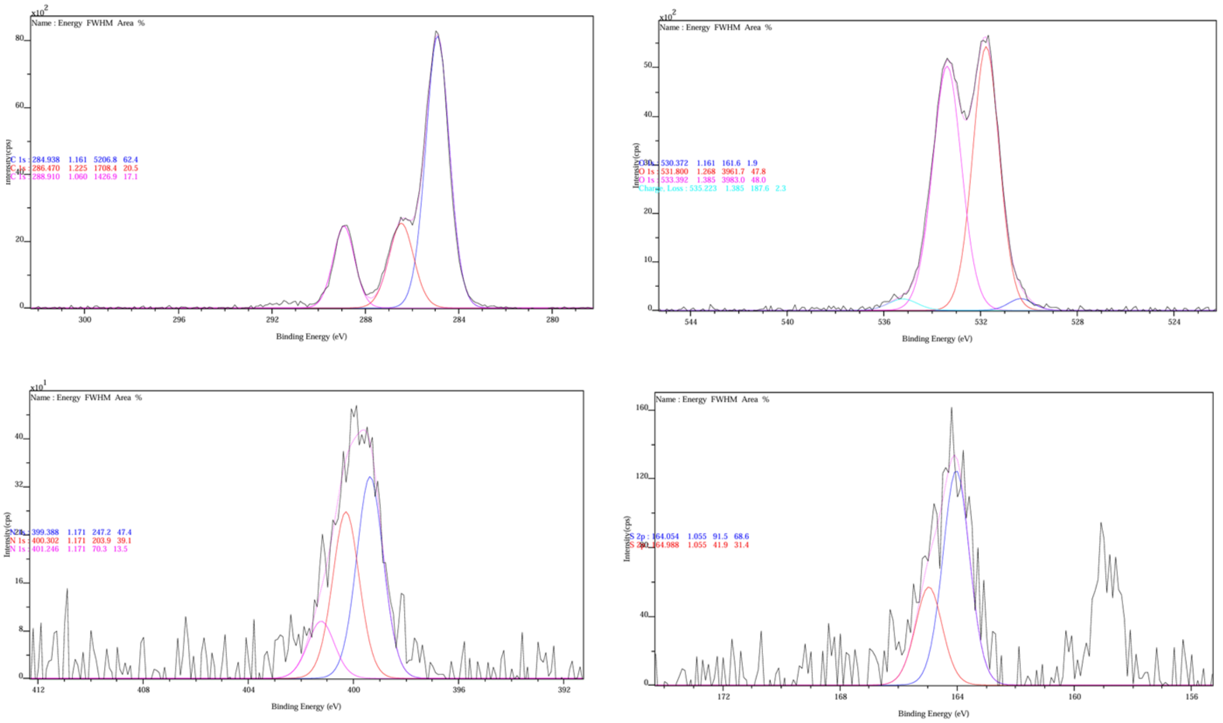

Figure S2. Raw XPS spectra of NSCQDs.

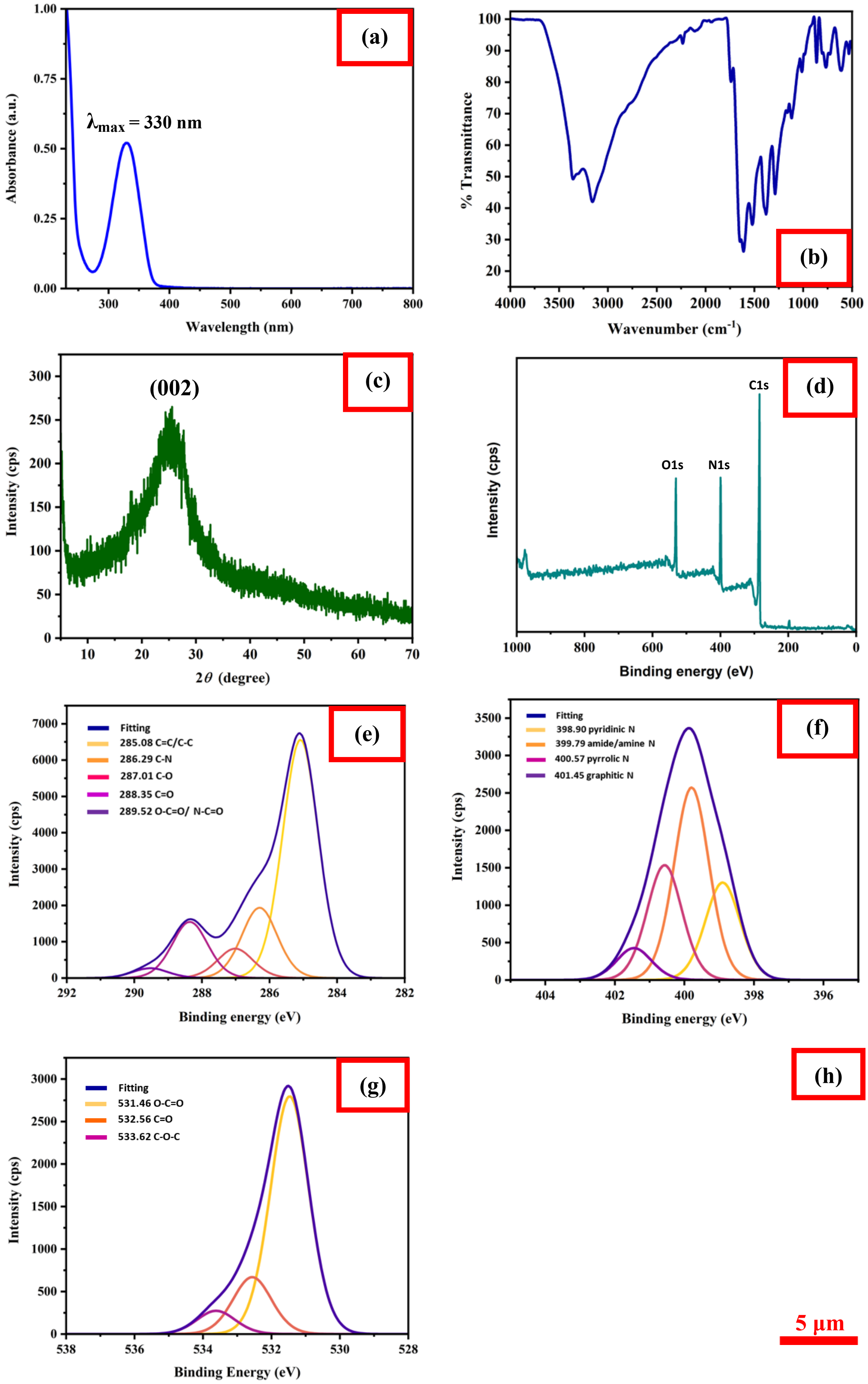

Figure S3. (a) UV-Vis spectrum of the synthesized **NCQDs**. (b) FTIR spectrum of the synthesized **NCQDs**. (c) XRD diffractogram of the synthesized **NCQDs**. (d) XPS survey spectra of **NCQDs**. The detailed (high-resolution) scans for (e) C 1s, (f) N 1s, (g) O 1s, and (h) typical SEM image of the **NCQDs**.

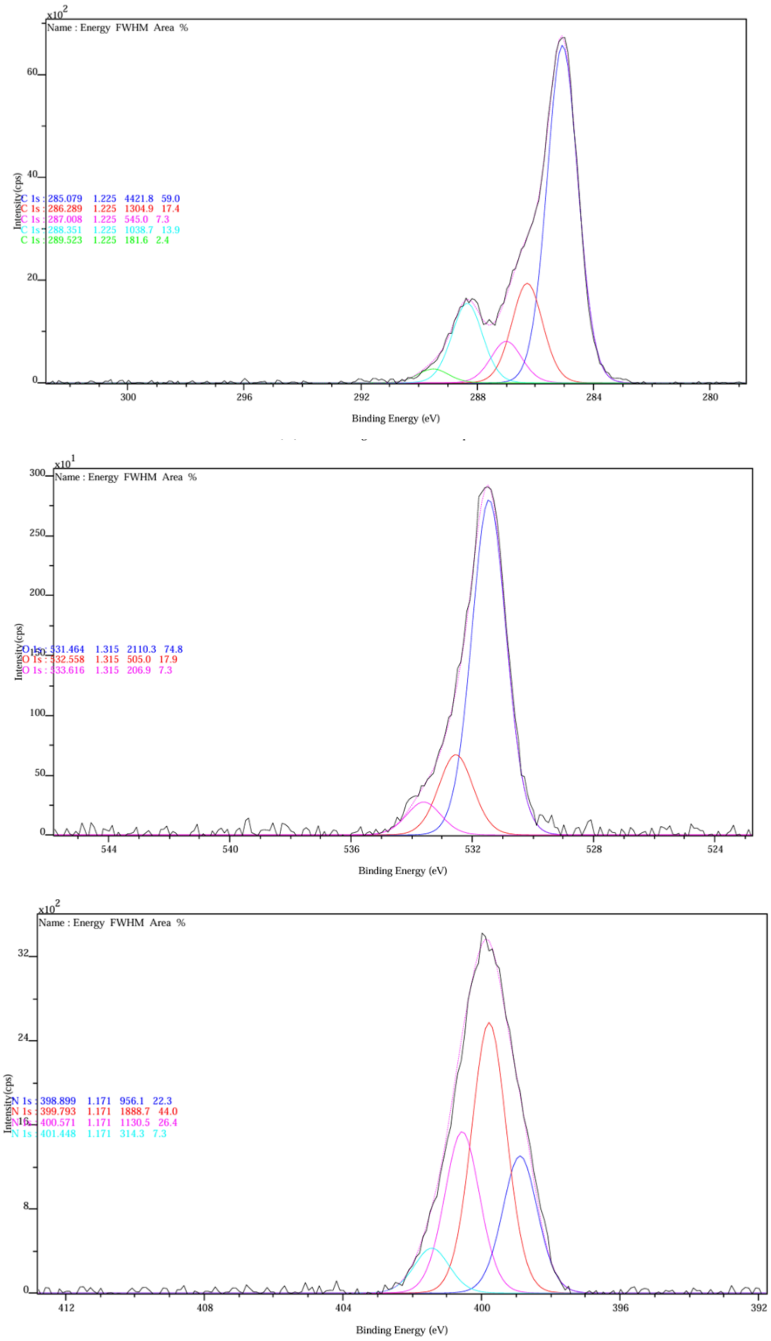

Figure S4. Raw XPS spectra of NCQDs.

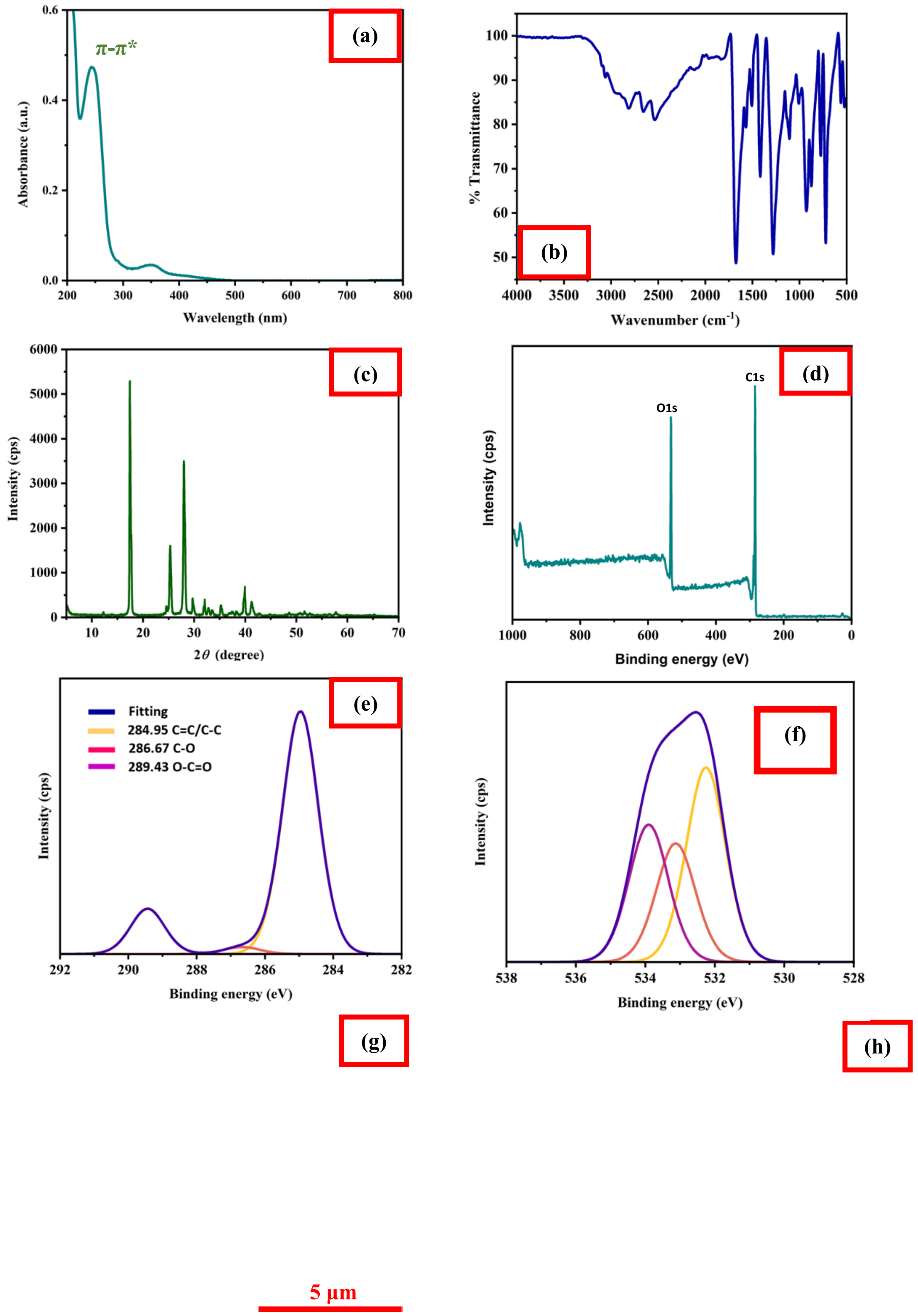

Figure S5. (a) UV-Vis spectrum of the synthesized **BCQDs**. (b) FTIR spectrum of the synthesized **BCQDs**. (c) XRD diffractogram of the synthesized **BCQDs**. (d) XPS survey spectra of **BCQDs**. The detailed (high-resolution) scans for (e) C 1s, (f) O 1s, (g) typical SEM image, and (h) EDS analysis of the **BCQDs**.

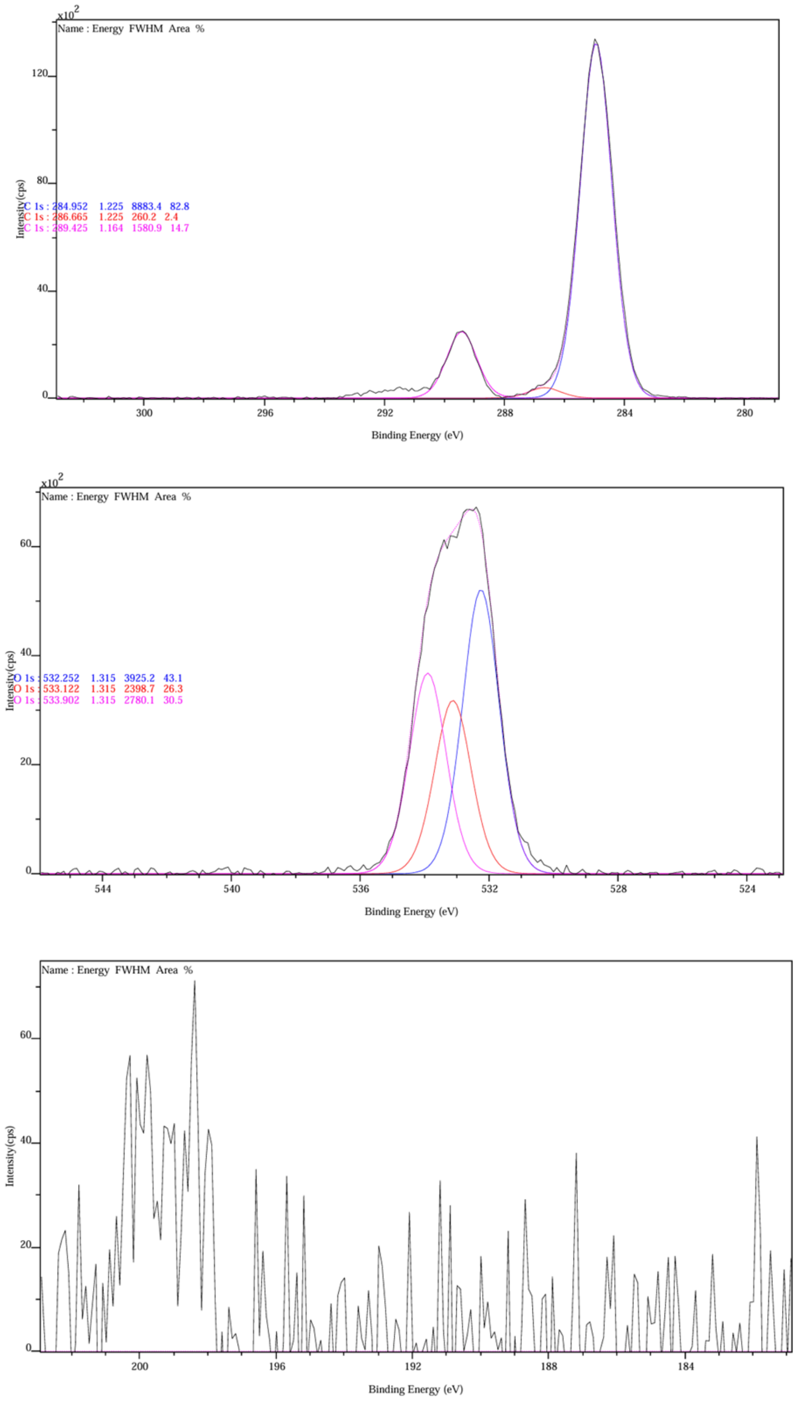

Figure S6. Raw XPS spectra of BCQDs.

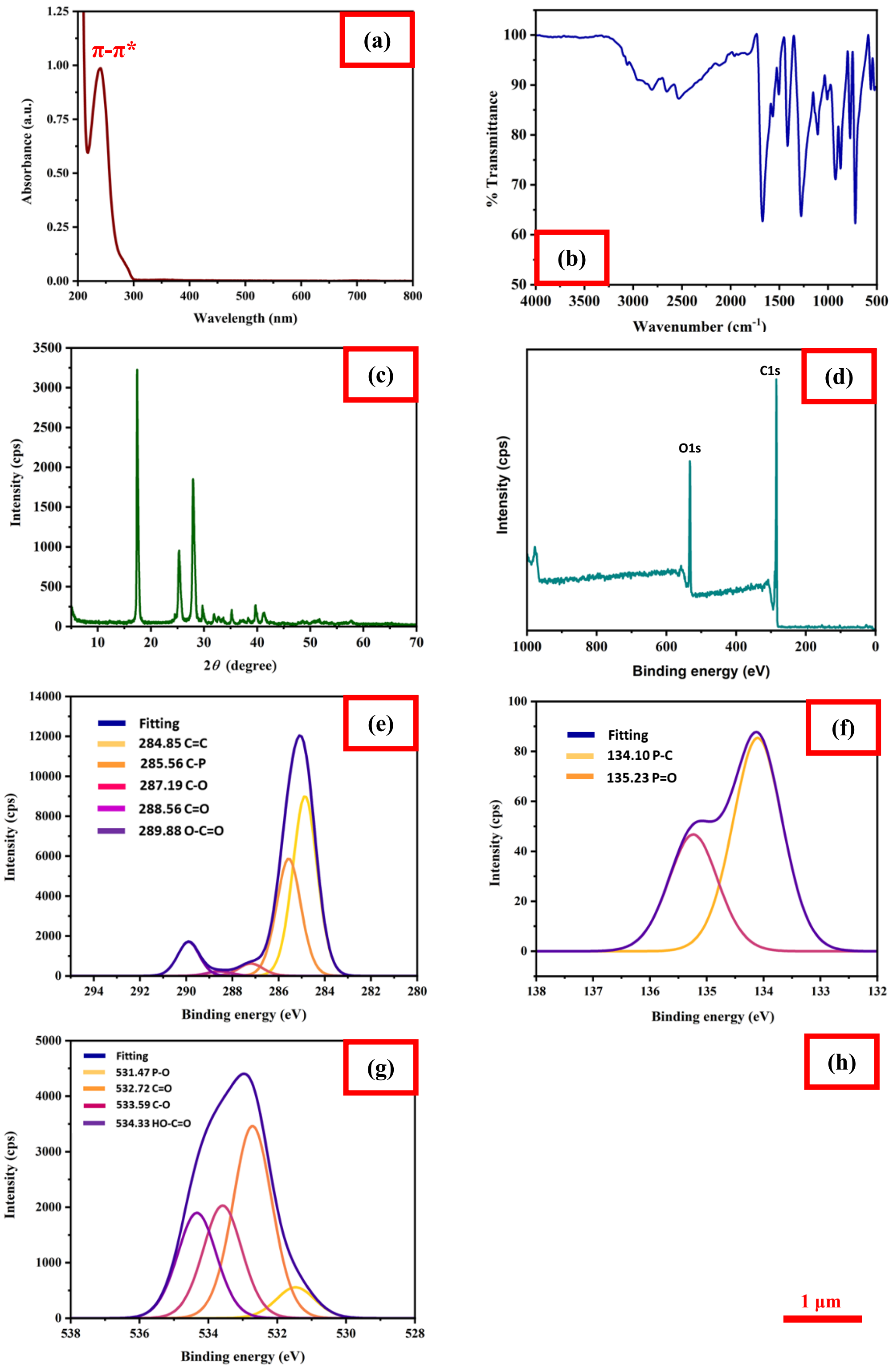

Figure S7. (a) UV-Vis spectrum of the synthesized **PCQDs**. (b) FTIR spectrum of the synthesized **PCQDs**. (c) XRD diffractogram of the synthesized **PCQDs**. (d) XPS survey spectra of **PCQDs**. The detailed (high-resolution) scans for (e) C 1s, (f) P 2p, (g) O 1s, and (h) typical SEM image of the **PCQDs**.

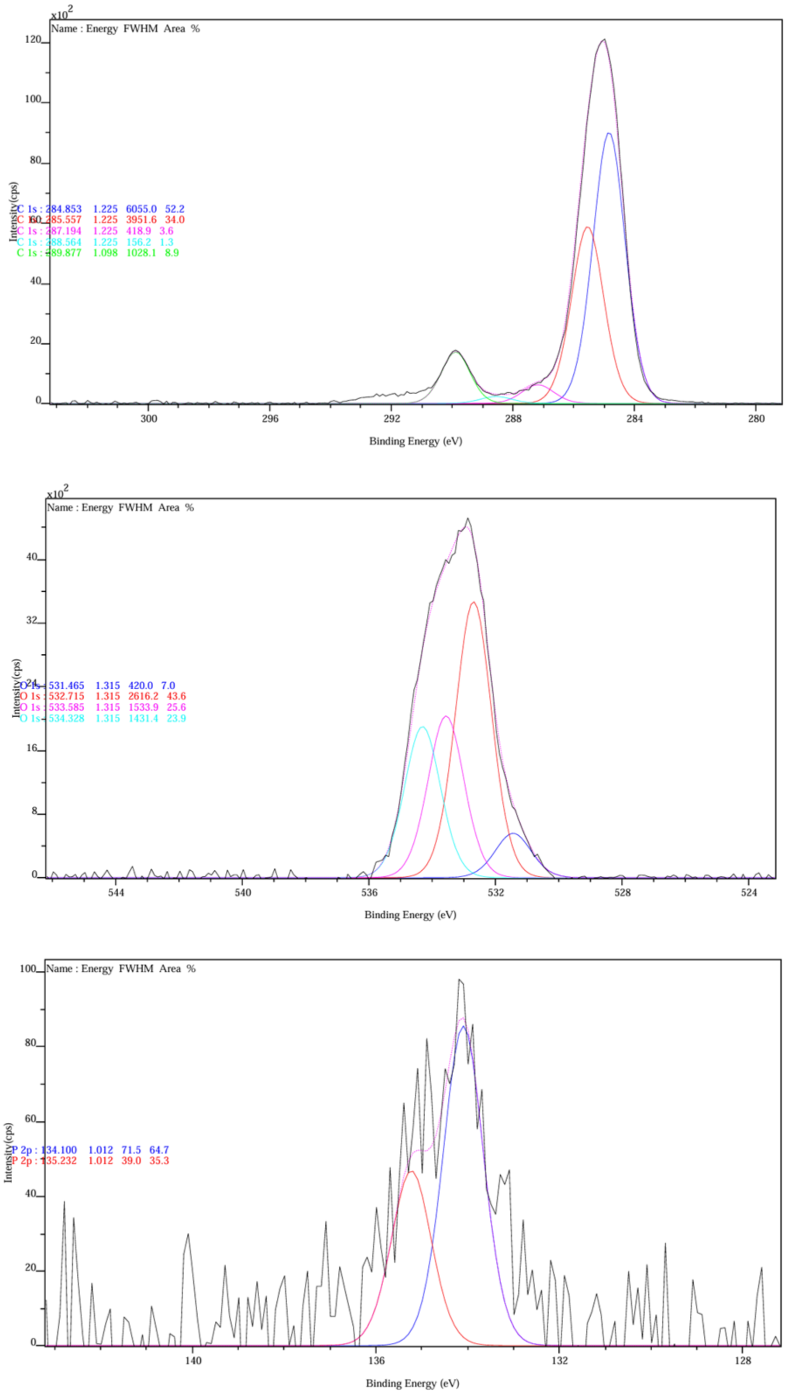

Figure S8. Raw XPS spectra of PCQDs.

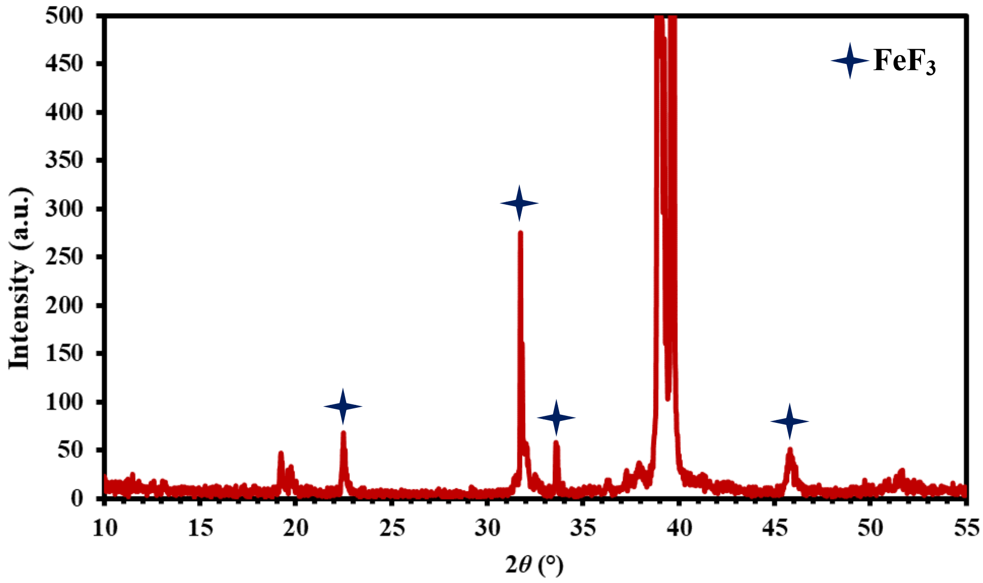

Figure S9. X-ray diffraction (XRD) patterns confirming the formation of a Fe–F product after the addition of fluoride ions (F^-) to the Fe^{3+}-quenched quantum dot system. The appearance of new diffraction peaks indicates the formation of a crystalline iron(III) fluoride phase, supporting the proposed mechanism in which F^- ions bind with Fe^{3+} to form an insoluble or weakly soluble complex. This process effectively removes Fe^{3+} from the surface of the quantum dots, enabling the restoration of their fluorescence signal ("off–on" behavior).

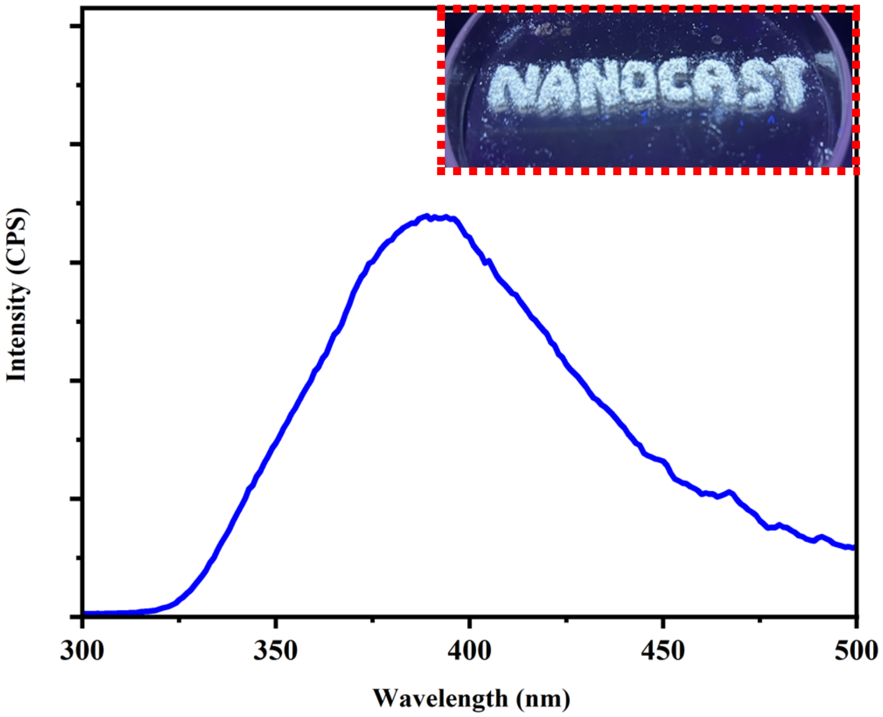

Figure S10. Solid state fluorescence spectra of **NCQDs** measured at 45°, front surface configuration. Excitation wavelength = 260 nm.

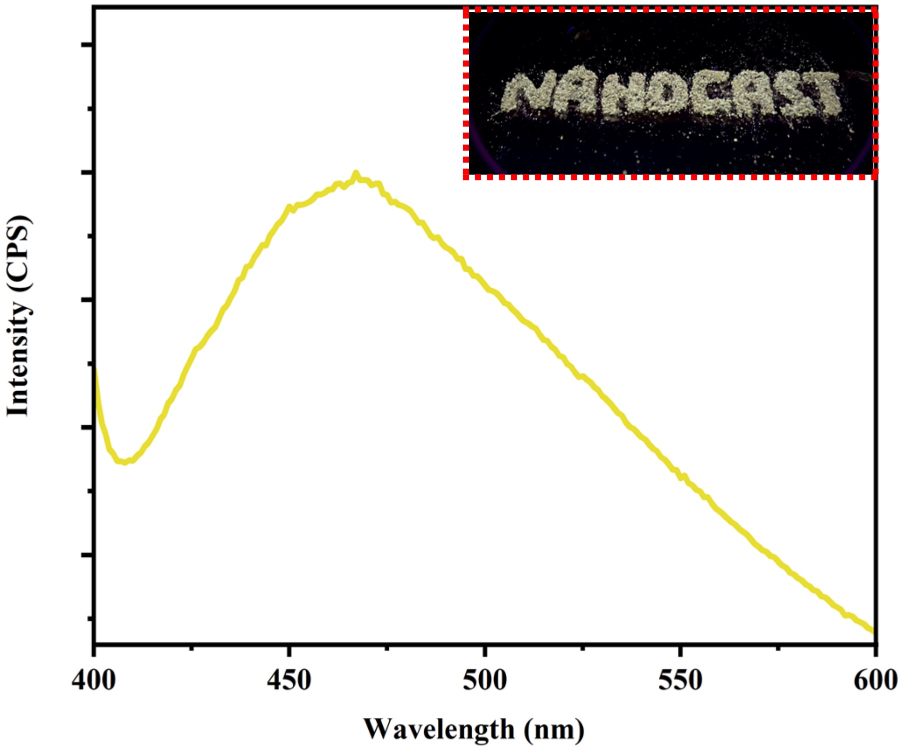

Figure S11. Solid state fluorescence spectra of **NSCQDs** measured at 45°, front surface configuration. Excitation wavelength = 389 nm

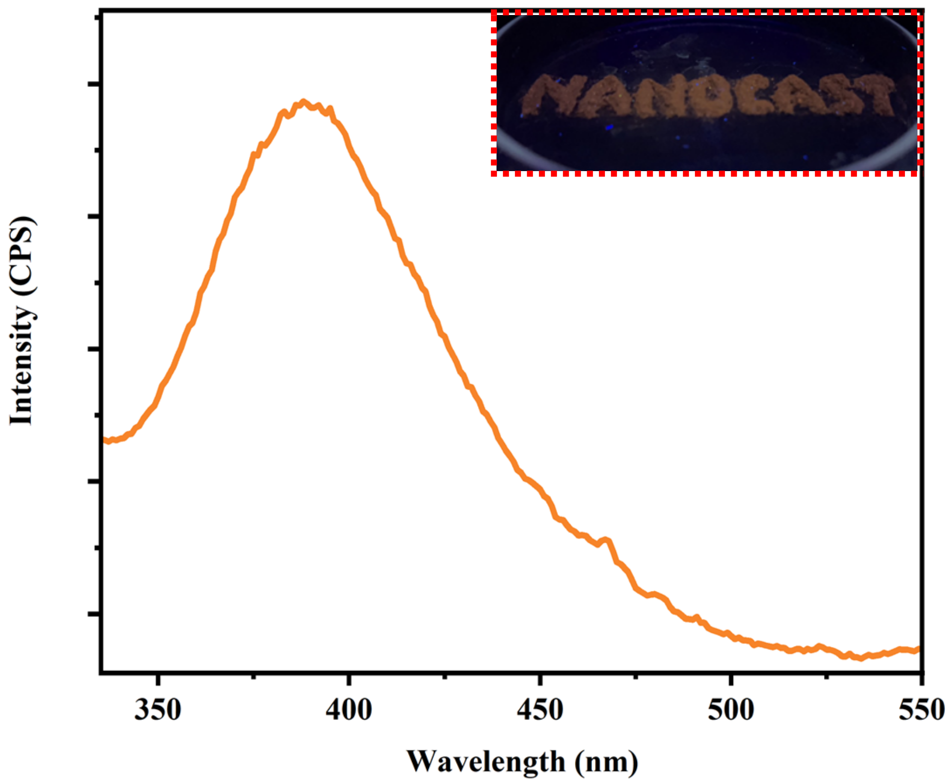

Figure S12. Solid state fluorescence spectra of **PCQDs** measured at 45°, front surface configuration. Excitation wavelength = 321 nm.
